# Bis{μ-2-[bis­(pyridin-2-yl)methyl­idene]hydrazinecarbothio­amidato}bis­[bromido­copper(II)] methanol disolvate

**DOI:** 10.1107/S1600536812005934

**Published:** 2012-02-29

**Authors:** Roji J. Kunnath, M. Sithambaresan, M. R. Prathapachandra Kurup, Aiswarya Natarajan, A. Ambili Aravindakshan

**Affiliations:** a Department of Applied Chemistry, Cochin University of Science and Technology, Kochi 682022, India; b Department of Chemistry, Faculty of Science, Eastern University, Sri Lanka, Chenkalady, Sri Lanka

## Abstract

In the centrosymmetric binuclear title compound, [Cu_2_Br_2_(C_12_H_10_N_5_S)_2_]·2CH_3_OH, the Cu^II^ ion adopts a slightly dis­torted square-pyramidal coordination geometry. The hydrazine carbothio­amide moiety and one of the pyridyl rings together adopt an almost planar arrangement, with a maximum deviation of 0.052 (4) Å for the C atom of the thio­urea moiety. There are two mol­ecules of methanol solvent per complex in the asymmetric unit. The nonconventional intra­molecular C—H⋯Br hydrogen bonds make the mol­ecule more rigid, whereas the conventional N—H⋯N and O—H⋯Br inter­molecular hydrogen-bonding inter­actions, supported with N—H⋯π inter­actions, establish a supra­molecular linkage among the mol­ecules in the crystal. An intermolecular C—H⋯O inter­action is also present.

## Related literature

For the biological applications of multinuclear copper complexes of hydrazinecarbothio­amide, see: Moubaraki *et al.* (1998 ▶); Khan *et al.* (1985 ▶). For the synthesis of the title compound, see: Philip *et al.* (2006 ▶). For related structures of dimeric copper complexes of hydrazinecarbothio­amide, see: Ainscough *et al.* (1991 ▶); Philip *et al.* (2005 ▶). For related literature, see: Duan *et al.* (1996 ▶).
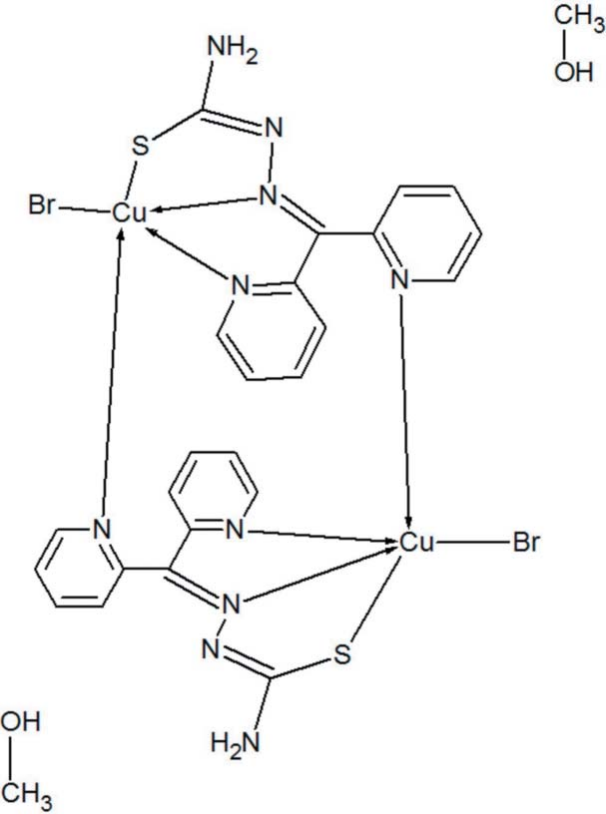


## Experimental

### 

#### Crystal data

[Cu_2_Br_2_(C_12_H_10_N_5_S)_2_]·2CH_4_O
*M* *_r_* = 863.62
Triclinic, 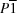

*a* = 8.3052 (7) Å
*b* = 9.2120 (7) Å
*c* = 11.0500 (9) Å
α = 68.341 (2)°
β = 79.127 (3)°
γ = 84.913 (2)°
*V* = 771.45 (11) Å^3^
*Z* = 1
Mo *K*α radiation
μ = 4.15 mm^−1^
*T* = 296 K
0.30 × 0.25 × 0.25 mm

#### Data collection

Bruker AXS Kappa APEXII CCD diffractometer
Absorption correction: multi-scan (*SADABS*; Bruker, 2004 ▶) *T*_min_ = 0.300, *T*_max_ = 0.354
11266 measured reflections
2688 independent reflections
2374 reflections with *I* > 2σ(*I*)
*R*_int_ = 0.065

#### Refinement

*R*[*F* ^2^ > 2σ(*F* ^2^)] = 0.034
*wR*(*F* ^2^) = 0.094
*S* = 1.08
2688 reflections
209 parameters
2 restraints
H atoms treated by a mixture of independent and constrained refinement
Δρ_max_ = 0.46 e Å^−3^
Δρ_min_ = −0.48 e Å^−3^

### 

Data collection: *APEX2* (Bruker, 2004 ▶); cell refinement: *APEX2*/*SAINT* (Bruker, 2004 ▶); data reduction: *SAINT*/*XPREP* (Bruker, 2004 ▶); program(s) used to solve structure: *SHELXS97* (Sheldrick, 2008 ▶); program(s) used to refine structure: *SHELXL97* (Sheldrick, 2008 ▶); molecular graphics: *ORTEP-3* (Farrugia, 1997 ▶) and *DIAMOND* (Brandenburg, 2010 ▶); software used to prepare material for publication: *SHELXL97* and *publCIF* (Westrip, 2010 ▶).

## Supplementary Material

Crystal structure: contains datablock(s) global, I. DOI: 10.1107/S1600536812005934/fj2511sup1.cif

Structure factors: contains datablock(s) I. DOI: 10.1107/S1600536812005934/fj2511Isup2.hkl

Additional supplementary materials:  crystallographic information; 3D view; checkCIF report

## Figures and Tables

**Table 1:** Hydrogen-bond geometry (Å, °) *Cg*4 is the centroid of the N2/C7–C11 ring.

*D*—H⋯*A*	*D*—H	H⋯*A*	*D*⋯*A*	*D*—H⋯*A*
O1—H1*A*⋯Br1^i^	0.82	2.58	3.396 (4)	178
N5—H5*A*⋯N4^ii^	0.84 (2)	2.17 (2)	3.006 (4)	177 (5)
C4—H4⋯O1^iii^	0.93	2.44	3.281 (5)	151
C11—H11⋯Br1^iv^	0.93	2.86	3.573 (4)	135
C1—H1⋯Br1	0.93	2.91	3.450 (4)	119
N5—H5*B*⋯*Cg*4^ii^	0.84 (2)	2.71 (4)	3.310 (4)	129 (3)

Symmetry codes: (i) 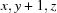; (ii) 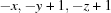; (iii) 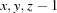; (iv) 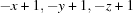.

**Fig. 1.**
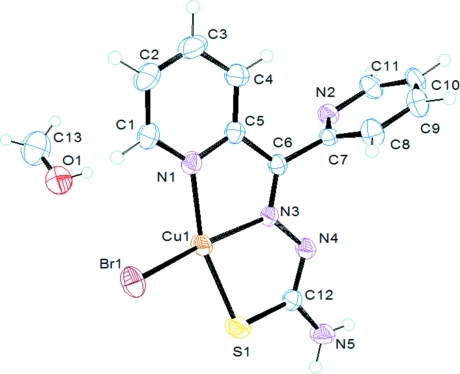
ORTEP view of the unique part of the Cu complex, drawn with 50% probability displacement ellipsoids for the non-H atoms.

**Fig. 2.**
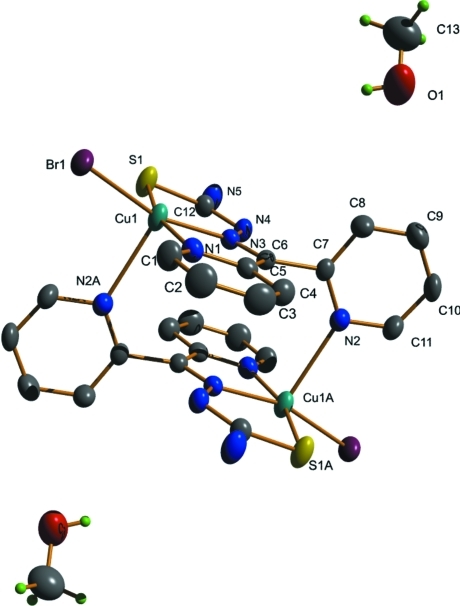
A view of the dimeric unit generated by the association of one of the pyridyl N of the ligand with the Cu atom of an adjacent molecule. The weak metal-axial ligand interaction is represented by dashed lines. Non-H atoms are drawn with 50% probability ellipsoids. The H atoms in the complex are omitted for clarity.

**Fig. 3.**
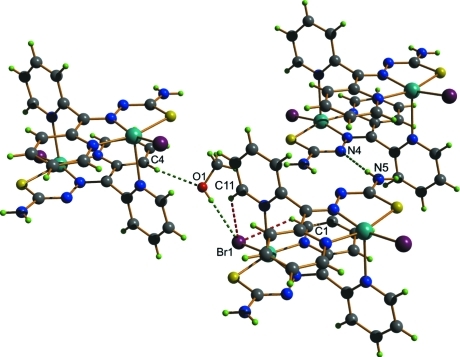
Hydrogen-bonding interactions showing an infinite chain in the crystal structure of [Cu2Br2(C12 H10 N5 S)2].2(C H3 OH).

## supplementary crystallographic information

### Comment

Hydrazinecarbothioamides have been reported to have a great variety of biological activity. In most cases, the metal complexes show more activity compared to their metal free ligands (Moubaraki *et al.*, 1998). Coupled systems of transition metal complexes are of special interest in various fields of science. The main reason probably is due to the phenomenon of interaction between metal centers lying at the crossover point of two widely separated areas, namely the physics of the magnetic materials and the role of polynuclear reaction sites in biological processes (Khan *et al.*, 1985).

The title complex [Cu_2_Br_2_(C_12_H_10_N_5_S)_2_].2(CH_3_OH) has a dimeric structure. The coordination geometry around each copper(II) ion is square pyramidal with a slight distortion (τ = 0.03). The S1 atom of the hydrazinecarbothioamide moiety, the imino N3 atom, pyridine N1 atom and the Br1 atom comprise the basal plane while the apical position is occupied by the N2A atom of the symmetry related half of the dimer with a longest bond length to the metal atom of 2.529 (3) Å. The hydrazinecarbothioamide moiety of the free ligand shows *E* configuration about the both C12–N4 and C6–N3 (Ainscough *et al.*, 1991; Philip *et al.*, 2005) whereas in the Cu^II^ complex the coordinated hydrazinecarbothioamide moiety has *E* configuration with respect to C6–N3 and *Z* configuration about C12–N4. The atoms coordinated to metal centre found to exist in *E* configuration having N3 and N1 atoms *cis* to each other with respect to C5—C6 bond. A unique part of the Cu^II^ complex and the dimeric unit generated by the association of the free pyridyl nitrogen with the Cu atom are shown along with the atom-labeling in Fig. 1 and 2 respectively. The two aromatic rings are twisted with a dihedral angle of 88.1 (2)° between the rings. The hydrazinecarbothioamide moiety and one of the pyridine ring comprising atoms C1—C6 and N1 are almost planar with maximum deviation of 0.052 (4) Å for the atom C12 of the ring. C12–S1 bond distance (1.727 (4) Å) is very close to the single bond (Duan *et al.*, 1996) which suggests that the ligand is coordinated in the thiolate form. This phenomenon could also be further confirmed by the coplanar nature of the NH_2_ group of the coordinated ligand with *sp*^2^ character wich facilitates an extended conjugation of the hydrazinecarbothioamide moiety with the aromatic rings.

The intramolecular non-classical hydrogen bonding interactions (C1–H1···Br1 and C11–H11···Br1), Table 1, makes the complex more rigid. The intermolecular hydrogen bonding interactions (classical and non-classical) establish a supramolecular 1-D network by linking the adjacent molecules through the methanol present in the lattice and N—H···N in parallel fashion as shown in Fig. 3. Packing of the molecules also involves many very weak π..π interactions with centroid-centroid distances in the range 3.707 (2)–5.778 (2). However, there is an N—H···π interaction between the hydrogen attached at N5 atom and one of the pyridyl ring comprising atoms from C7—C11 and N2 of another molecule and also a lone-pair···π interaction between the Br1 atom and two different chelate rings comprising atoms Cu1, S1, C12, N3, N4 and Cu1, N1, N3, C5, C6.

### Experimental

The title complex was prepared by adapting a reported procedure (Philip *et al.*, 2006) by refluxing a mixture of methanolic solutions of 2-[di(pyridin-2-yl)methylidene]hydrazinecarbothioamide (2.573 g, 10 mmol) and CuBr_2_ (2.230 g, 10 mmol) for four hours. Black colored crystals were collected, washed with few drops of methanol and dried over P_4_O_10_*in vacuo*. Single crystals of the title complex suitable for X-ray analysis were obtained by slow evaporation from its methanolic solution.

### Refinement

All H atoms on C were placed in calculated positions, guided by difference maps, with C—H bond distances 0.93–0.96 Å. H atoms were assigned as *U*_iso_=1.2 *U*_eq_ (1.5 for Me). N5—H5A and N5—H5B H atoms were located from difference maps and restrained using *DFIX* instructions. The O1—H1A (0.82 Å) hydrogen of the methanol solvent is also placed in calculated position guided by difference maps.

### Crystal data

**Table:**

[Cu_2_Br_2_(C_12_H_10_N_5_S)_2_]·2CH_4_O	*Z* = 1
*M**_r_* = 863.62	*F*(000) = 430.0
Triclinic, *P*1	*D*_x_ = 1.859 Mg m^−^^3^
Hall symbol: -P 1	Mo *K*α radiation, λ = 0.71073 Å
*a* = 8.3052 (7) Å	Cell parameters from 7050 reflections
*b* = 9.2120 (7) Å	θ = 2.4–28.3°
*c* = 11.0500 (9) Å	µ = 4.15 mm^−^^1^
α = 68.341 (2)°	*T* = 296 K
β = 79.127 (3)°	Block, black
γ = 84.913 (2)°	0.30 × 0.25 × 0.25 mm
*V* = 771.45 (11) Å^3^	

### Data collection

**Table:**

Bruker AXS Kappa APEXII CCD diffractometer	2688 independent reflections
Radiation source: fine-focus sealed tube	2374 reflections with *I* > 2σ(*I*)
Graphite monochromator	*R*_int_ = 0.065
Detector resolution: 8.33 pixels mm^-1^	θ_max_ = 25.0°, θ_min_ = 2.4°
ω and φ scan	*h* = −9→9
Absorption correction: multi-scan (*SADABS*; Bruker, 2004)	*k* = −9→10
*T*_min_ = 0.300, *T*_max_ = 0.354	*l* = −13→13
11266 measured reflections	

### Refinement

**Table:**

Refinement on *F*^2^	Primary atom site location: structure-invariant direct methods
Least-squares matrix: full	Secondary atom site location: difference Fourier map
*R*[*F*^2^ > 2σ(*F*^2^)] = 0.034	Hydrogen site location: inferred from neighbouring sites
*wR*(*F*^2^) = 0.094	H atoms treated by a mixture of independent and constrained refinement
*S* = 1.08	*w* = 1/[σ^2^(*F*_o_^2^) + (0.0351*P*)^2^ + 0.9038*P*] where *P* = (*F*_o_^2^ + 2*F*_c_^2^)/3
2688 reflections	(Δ/σ)_max_ = 0.001
209 parameters	Δρ_max_ = 0.46 e Å^−^^3^
2 restraints	Δρ_min_ = −0.48 e Å^−^^3^

### Special details

**Table:**

Geometry. All e.s.d.'s (except the e.s.d. in the dihedral angle between two l.s. planes) are estimated using the full covariance matrix. The cell e.s.d.'s are taken into account individually in the estimation of e.s.d.'s in distances, angles and torsion angles; correlations between e.s.d.'s in cell parameters are only used when they are defined by crystal symmetry. An approximate (isotropic) treatment of cell e.s.d.'s is used for estimating e.s.d.'s involving l.s. planes.
Refinement. Refinement of *F*^2^ against ALL reflections. The weighted *R*-factor *wR* and goodness of fit *S* are based on *F*^2^, conventional *R*-factors *R* are based on *F*, with *F* set to zero for negative *F*^2^. The threshold expression of *F*^2^ > σ(*F*^2^) is used only for calculating *R*-factors(gt) *etc*. and is not relevant to the choice of reflections for refinement. *R*-factors based on *F*^2^ are statistically about twice as large as those based on *F*, and *R*- factors based on ALL data will be even larger.

### Fractional atomic coordinates and isotropic or equivalent isotropic displacement parameters (Å^2^)

**Table:**

	*x*	*y*	*z*	*U*_iso_*/*U*_eq_	
Br1	0.58511 (5)	−0.08863 (4)	0.69095 (4)	0.04060 (15)	
Cu1	0.47922 (5)	0.17346 (5)	0.59664 (4)	0.03332 (15)	
S1	0.22214 (12)	0.12075 (11)	0.70508 (10)	0.0434 (3)	
O1	0.7785 (5)	0.7271 (5)	0.9538 (4)	0.0823 (12)	
H1A	0.7308	0.7735	0.8912	0.123*	
N1	0.6759 (3)	0.2576 (3)	0.4581 (3)	0.0299 (6)	
N2	0.4272 (3)	0.7176 (3)	0.2495 (3)	0.0302 (6)	
N3	0.3754 (3)	0.3618 (3)	0.4794 (3)	0.0238 (6)	
N4	0.2129 (3)	0.3997 (3)	0.5030 (3)	0.0294 (6)	
N5	−0.0218 (4)	0.3210 (4)	0.6483 (3)	0.0417 (8)	
C1	0.8282 (4)	0.2004 (5)	0.4516 (4)	0.0379 (9)	
H1	0.8526	0.1102	0.5197	0.045*	
C2	0.9519 (5)	0.2690 (5)	0.3480 (4)	0.0465 (10)	
H2	1.0579	0.2269	0.3470	0.056*	
C3	0.9154 (5)	0.3999 (5)	0.2473 (4)	0.0445 (10)	
H3	0.9964	0.4475	0.1758	0.053*	
C4	0.7571 (5)	0.4617 (5)	0.2518 (4)	0.0360 (8)	
H4	0.7305	0.5510	0.1838	0.043*	
C5	0.6401 (4)	0.3882 (4)	0.3587 (3)	0.0268 (7)	
C6	0.4688 (4)	0.4423 (4)	0.3730 (3)	0.0248 (7)	
C7	0.4119 (4)	0.5710 (4)	0.2597 (3)	0.0248 (7)	
C8	0.3584 (5)	0.5319 (4)	0.1652 (4)	0.0384 (9)	
H8	0.3479	0.4276	0.1771	0.046*	
C9	0.3207 (5)	0.6484 (5)	0.0535 (4)	0.0433 (10)	
H9	0.2855	0.6248	−0.0122	0.052*	
C10	0.3360 (5)	0.7995 (4)	0.0412 (4)	0.0377 (9)	
H10	0.3116	0.8812	−0.0334	0.045*	
C11	0.3880 (5)	0.8296 (4)	0.1402 (4)	0.0372 (9)	
H11	0.3965	0.9332	0.1310	0.045*	
C12	0.1364 (4)	0.2936 (4)	0.6108 (3)	0.0288 (7)	
C13	0.8997 (8)	0.8191 (8)	0.9536 (7)	0.0809 (17)	
H13A	0.8666	0.8600	1.0230	0.121*	
H13B	0.9186	0.9039	0.8700	0.121*	
H13C	0.9988	0.7578	0.9675	0.121*	
H5A	−0.074 (5)	0.401 (4)	0.608 (4)	0.058 (14)*	
H5B	−0.078 (5)	0.261 (4)	0.718 (3)	0.053 (14)*	

### Atomic displacement parameters (Å^2^)

**Table:**

	*U*^11^	*U*^22^	*U*^33^	*U*^12^	*U*^13^	*U*^23^
Br1	0.0512 (3)	0.0267 (2)	0.0425 (3)	0.01050 (16)	−0.01669 (19)	−0.00917 (17)
Cu1	0.0283 (3)	0.0284 (3)	0.0318 (3)	0.00663 (18)	−0.00458 (19)	0.00051 (19)
S1	0.0334 (5)	0.0364 (5)	0.0404 (6)	0.0031 (4)	−0.0012 (4)	0.0062 (4)
O1	0.070 (3)	0.089 (3)	0.072 (3)	−0.014 (2)	−0.023 (2)	−0.002 (2)
N1	0.0262 (15)	0.0315 (16)	0.0310 (16)	0.0048 (12)	−0.0072 (12)	−0.0103 (13)
N2	0.0306 (16)	0.0280 (16)	0.0283 (16)	0.0002 (12)	−0.0047 (13)	−0.0061 (12)
N3	0.0202 (14)	0.0251 (14)	0.0256 (14)	0.0027 (11)	−0.0054 (11)	−0.0084 (11)
N4	0.0260 (15)	0.0281 (15)	0.0283 (15)	0.0045 (12)	−0.0048 (12)	−0.0045 (12)
N5	0.0282 (17)	0.042 (2)	0.0375 (19)	0.0030 (15)	0.0036 (15)	0.0003 (16)
C1	0.0269 (19)	0.040 (2)	0.042 (2)	0.0102 (16)	−0.0082 (17)	−0.0107 (17)
C2	0.028 (2)	0.052 (3)	0.055 (3)	0.0104 (18)	−0.0033 (19)	−0.018 (2)
C3	0.029 (2)	0.053 (3)	0.047 (2)	−0.0023 (17)	0.0049 (18)	−0.018 (2)
C4	0.034 (2)	0.037 (2)	0.033 (2)	−0.0006 (16)	−0.0025 (16)	−0.0095 (16)
C5	0.0275 (18)	0.0287 (17)	0.0272 (18)	0.0022 (13)	−0.0070 (14)	−0.0128 (14)
C6	0.0267 (18)	0.0222 (16)	0.0251 (17)	0.0011 (13)	−0.0067 (14)	−0.0074 (14)
C7	0.0202 (16)	0.0259 (17)	0.0229 (16)	0.0010 (13)	−0.0013 (13)	−0.0042 (13)
C8	0.049 (2)	0.0285 (19)	0.040 (2)	0.0026 (16)	−0.0159 (18)	−0.0121 (16)
C9	0.050 (2)	0.049 (2)	0.034 (2)	0.0027 (19)	−0.0166 (18)	−0.0155 (18)
C10	0.042 (2)	0.037 (2)	0.0258 (19)	0.0055 (16)	−0.0116 (16)	−0.0002 (15)
C11	0.047 (2)	0.0259 (18)	0.033 (2)	0.0012 (16)	−0.0087 (17)	−0.0031 (15)
C12	0.0238 (17)	0.0317 (18)	0.0287 (18)	0.0032 (14)	−0.0054 (14)	−0.0087 (15)
C13	0.077 (4)	0.087 (4)	0.092 (4)	0.006 (3)	−0.024 (3)	−0.045 (4)

### Geometric parameters (Å, º)

**Table:**

Br1—Cu1	2.4084 (5)	C2—C3	1.363 (6)
Cu1—N3	1.982 (3)	C2—H2	0.9300
Cu1—N1	2.005 (3)	C3—C4	1.385 (5)
Cu1—S1	2.2404 (11)	C3—H3	0.9300
S1—C12	1.727 (3)	C4—C5	1.376 (5)
O1—C13	1.371 (7)	C4—H4	0.9300
O1—H1A	0.8200	C5—C6	1.464 (5)
N1—C1	1.326 (4)	C6—C7	1.492 (4)
N1—C5	1.351 (4)	C7—C8	1.378 (5)
N2—C7	1.329 (4)	C8—C9	1.372 (5)
N2—C11	1.341 (5)	C8—H8	0.9300
N3—C6	1.284 (4)	C9—C10	1.363 (6)
N3—N4	1.365 (4)	C9—H9	0.9300
N4—C12	1.320 (4)	C10—C11	1.373 (5)
N5—C12	1.333 (5)	C10—H10	0.9300
N5—H5A	0.837 (19)	C11—H11	0.9300
N5—H5B	0.843 (19)	C13—H13A	0.9600
C1—C2	1.379 (6)	C13—H13B	0.9600
C1—H1	0.9300	C13—H13C	0.9600
			
N3—Cu1—N1	81.17 (11)	N1—C5—C4	121.6 (3)
N3—Cu1—S1	83.78 (8)	N1—C5—C6	115.1 (3)
N1—Cu1—S1	163.80 (9)	C4—C5—C6	123.3 (3)
N3—Cu1—Br1	162.00 (8)	N3—C6—C5	115.6 (3)
N1—Cu1—Br1	97.02 (8)	N3—C6—C7	125.0 (3)
S1—Cu1—Br1	95.45 (3)	C5—C6—C7	118.9 (3)
C12—S1—Cu1	95.59 (12)	N2—C7—C8	123.3 (3)
C13—O1—H1A	109.5	N2—C7—C6	118.5 (3)
C1—N1—C5	118.6 (3)	C8—C7—C6	118.1 (3)
C1—N1—Cu1	128.9 (3)	C9—C8—C7	119.3 (3)
C5—N1—Cu1	112.5 (2)	C9—C8—H8	120.3
C7—N2—C11	116.4 (3)	C7—C8—H8	120.3
C6—N3—N4	121.0 (3)	C10—C9—C8	118.2 (4)
C6—N3—Cu1	115.6 (2)	C10—C9—H9	120.9
N4—N3—Cu1	123.3 (2)	C8—C9—H9	120.9
C12—N4—N3	111.1 (3)	C9—C10—C11	119.2 (3)
C12—N5—H5A	124 (3)	C9—C10—H10	120.4
C12—N5—H5B	123 (3)	C11—C10—H10	120.4
H5A—N5—H5B	113 (4)	N2—C11—C10	123.5 (4)
N1—C1—C2	122.8 (4)	N2—C11—H11	118.2
N1—C1—H1	118.6	C10—C11—H11	118.2
C2—C1—H1	118.6	N4—C12—N5	117.0 (3)
C3—C2—C1	118.6 (4)	N4—C12—S1	125.8 (3)
C3—C2—H2	120.7	N5—C12—S1	117.2 (3)
C1—C2—H2	120.7	O1—C13—H13A	109.5
C2—C3—C4	119.7 (4)	O1—C13—H13B	109.5
C2—C3—H3	120.2	H13A—C13—H13B	109.5
C4—C3—H3	120.2	O1—C13—H13C	109.5
C5—C4—C3	118.7 (4)	H13A—C13—H13C	109.5
C5—C4—H4	120.7	H13B—C13—H13C	109.5
C3—C4—H4	120.7		
			
N3—Cu1—S1—C12	−4.96 (14)	C3—C4—C5—N1	−0.5 (5)
N1—Cu1—S1—C12	−26.6 (3)	C3—C4—C5—C6	−179.3 (3)
Br1—Cu1—S1—C12	−166.89 (12)	N4—N3—C6—C5	−179.2 (3)
N3—Cu1—N1—C1	179.6 (3)	Cu1—N3—C6—C5	−3.8 (4)
S1—Cu1—N1—C1	−158.5 (3)	N4—N3—C6—C7	−6.7 (5)
Br1—Cu1—N1—C1	−18.4 (3)	Cu1—N3—C6—C7	168.7 (2)
N3—Cu1—N1—C5	−2.2 (2)	N1—C5—C6—N3	1.9 (4)
S1—Cu1—N1—C5	19.7 (5)	C4—C5—C6—N3	−179.3 (3)
Br1—Cu1—N1—C5	159.8 (2)	N1—C5—C6—C7	−171.0 (3)
N1—Cu1—N3—C6	3.3 (2)	C4—C5—C6—C7	7.8 (5)
S1—Cu1—N3—C6	−170.7 (2)	C11—N2—C7—C8	−0.3 (5)
Br1—Cu1—N3—C6	−82.2 (3)	C11—N2—C7—C6	174.3 (3)
N1—Cu1—N3—N4	178.6 (3)	N3—C6—C7—N2	99.6 (4)
S1—Cu1—N3—N4	4.6 (2)	C5—C6—C7—N2	−88.2 (4)
Br1—Cu1—N3—N4	93.1 (3)	N3—C6—C7—C8	−85.5 (4)
C6—N3—N4—C12	173.7 (3)	C5—C6—C7—C8	86.7 (4)
Cu1—N3—N4—C12	−1.3 (4)	N2—C7—C8—C9	1.0 (6)
C5—N1—C1—C2	0.3 (6)	C6—C7—C8—C9	−173.6 (3)
Cu1—N1—C1—C2	178.4 (3)	C7—C8—C9—C10	−0.8 (6)
N1—C1—C2—C3	−1.0 (6)	C8—C9—C10—C11	−0.1 (6)
C1—C2—C3—C4	0.8 (6)	C7—N2—C11—C10	−0.7 (5)
C2—C3—C4—C5	−0.1 (6)	C9—C10—C11—N2	0.9 (6)
C1—N1—C5—C4	0.4 (5)	N3—N4—C12—N5	176.8 (3)
Cu1—N1—C5—C4	−178.0 (3)	N3—N4—C12—S1	−4.8 (4)
C1—N1—C5—C6	179.3 (3)	Cu1—S1—C12—N4	7.2 (3)
Cu1—N1—C5—C6	0.9 (3)	Cu1—S1—C12—N5	−174.4 (3)

### Hydrogen-bond geometry (Å, º)

Cg4 is the centroid of the N2/C7–C11 ring.

**Table:**

*D*—H···*A*	*D*—H	H···*A*	*D*···*A*	*D*—H···*A*
O1—H1*A*···Br1^i^	0.82	2.58	3.396 (4)	178
N5—H5*A*···N4^ii^	0.84 (2)	2.17 (2)	3.006 (4)	177 (5)
C4—H4···O1^iii^	0.93	2.44	3.281 (5)	151
C11—H11···Br1^iv^	0.93	2.86	3.573 (4)	135
C1—H1···Br1	0.93	2.91	3.450 (4)	119
N5—H5*B*···*Cg*4^ii^	0.84 (2)	2.71 (4)	3.310 (4)	129 (3)

Symmetry codes: (i) *x*, *y*+1, *z*; (ii) −*x*, −*y*+1, −*z*+1; (iii) *x*, *y*, *z*−1; (iv) −*x*+1, −*y*+1, −*z*+1.
